# Prevalence of evidence of inconsistency and its association with network structural characteristics in 201 published networks of interventions

**DOI:** 10.1186/s12874-021-01401-y

**Published:** 2021-10-25

**Authors:** Areti Angeliki Veroniki, Sofia Tsokani, Ian R. White, Guido Schwarzer, Gerta Rücker, Dimitris Mavridis, Julian P. T. Higgins, Georgia Salanti

**Affiliations:** 1 Knowledge Translation Program, Li Ka Shing Knowledge Institute, St. Michael’s Hospital, 209 Victoria Street, Toronto, Ontario M5B 1W8 Canada; 2 Institute of Reproductive and Developmental Biology, Department of Surgery & Cancer, Faculty of Medicine, Imperial College, London, UK; 3 Department of Primary Education, School of Education University of Ioannina, Ioannina, Greece; 4 Medical Research Council Clinical Trials Unit (MRC CTU), Institute of Clinical Trials and Methodology, University College London, 90 High Holborn 2nd Floor, London, WC1V 6LJ UK; 5 Institute of Medical Biometry and Statistics, Faculty of Medicine and Medical Center, University of Freiburg, Stefan-Meier-Strasse 26, 79104 Freiburg, Germany; 6 Paris Descartes University, Sorbonne Paris Cité, Faculté de Médecine, Paris, France; 7 School of Social and Community Medicine, University of Bristol, Canynge Hall, 39 Whatley Road, Bristol, BS8 2PS UK; 8 Institute of Social and Preventive Medicine (ISPM), University of Bern, Mittelstrasse 43, CH-3012 Bern, Switzerland

**Keywords:** Indirect evidence, Network meta-analysis, Chi-squared test, Between-study variance, Incoherence

## Abstract

**Background:**

Network meta-analysis (NMA) has attracted growing interest in evidence-based medicine. Consistency between different sources of evidence is fundamental to the reliability of the NMA results. The purpose of the present study was to estimate the prevalence of evidence of inconsistency and describe its association with different NMA characteristics.

**Methods:**

We updated our collection of NMAs with articles published up to July 2018. We included networks with randomised clinical trials, at least four treatment nodes, at least one closed loop, a dichotomous primary outcome, and available arm-level data. We assessed consistency using the design-by-treatment interaction (DBT) model and testing all the inconsistency parameters globally through the Wald-type chi-squared test statistic. We estimated the prevalence of evidence of inconsistency and its association with different network characteristics (e.g., number of studies, interventions, intervention comparisons, loops). We evaluated the influence of the network characteristics on the DBT *p*-value via a multivariable regression analysis and the estimated Pearson correlation coefficients. We also evaluated heterogeneity in NMA (consistency) and DBT (inconsistency) random-effects models.

**Results:**

We included 201 published NMAs. The *p*-value of the design-by-treatment interaction (DBT) model was lower than 0.05 in 14% of the networks and lower than 0.10 in 20% of the networks. Networks including many studies and comparing few interventions were more likely to have small DBT *p*-values (less than 0.10), which is probably because they yielded more precise estimates and power to detect differences between designs was higher. In the presence of inconsistency (DBT *p*-value lower than 0.10), the consistency model displayed higher heterogeneity than the DBT model.

**Conclusions:**

Our findings show that inconsistency was more frequent than what would be expected by chance, suggesting that researchers should devote more resources to exploring how to mitigate inconsistency. The results of this study highlight the need to develop strategies to detect inconsistency (because of the relatively high prevalence of evidence of inconsistency in published networks), and particularly in cases where the existing tests have low power.

**Supplementary Information:**

The online version contains supplementary material available at 10.1186/s12874-021-01401-y.

## Background

Network meta-analysis (NMA) is a useful approach for exploring effects of multiple interventions by simultaneously synthesizing direct and indirect evidence, and in recent years the number of published NMAs has grown continually [1, 2]. The reliability of inferences from NMA depends on the comparability of studies evaluating the multiple interventions of interest [3–5]. NMA results are valid only when the transitivity assumption holds, i.e., the distribution of effect modifiers is similar across intervention comparisons. Lack of transitivity can create statistical disagreement between the information coming from direct and indirect sources of evidence, that is inconsistency. The notion of inconsistency also refers to the disagreement between evidence coming from different designs (i.e., studies of different sets of interventions across studies). For example, a study comparing interventions A vs. B vs. C corresponds to a different design from study comparing A vs. B interventions. Several statistical methods exist to assess inconsistency in the network as a whole or locally on specific comparisons or loops of evidence (i.e., paths in the network of interventions that start and end at the same node) [6–12]. To date, the design-by-treatment interaction (DBT) model is the only method that both provides a global assessment of inconsistency for a network and is insensitive to the parameterization of studies with multiple arms [6, 8].

The majority of NMAs published in the medical literature in the recent years examine whether the prerequisite assumptions in NMA are met [1, 2, 13, 14]. However, a number of reviews still combine direct and indirect evidence in a network of interventions without evaluating the condition of consistency or despite evidence of inconsistency [15, 16]. Empirical findings for dichotomous outcomes suggest that inconsistency is present in one in ten loops of evidence and in one in eight networks [10]. It is encouraging though that authors of NMAs increasingly discuss transitivity and/or inconsistency (0% in 2005 vs 86% in 2015), and use appropriate methods to test for inconsistency (14% in 2006 vs 74% in 2015) [2]. Another important consideration when conducting an NMA is that there is an inverse association between heterogeneity and statistical power to detect inconsistency [3]. The larger the heterogeneity, the less precise the direct and indirect estimates are, and hence statistical inconsistency may not be evident even when it is present. Empirical evidence using 40 networks of interventions suggested that increased heterogeneity was associated with low detection rates of inconsistency, and that the consistency model displayed higher heterogeneity than the inconsistency model [10]. Also, the same study showed that the choice of the heterogeneity estimator (e.g., DerSimonian and Laird, maximum likelihood, restricted maximum likelihood, Paule and Mandel) can influence inferences about inconsistency particularly when few studies are available. In general inconsistency tests have low power to detect inconsistency, and power may vary depending on the network’s characteristics (e.g., number of participants and studies) [17].

The purpose of the present study was to estimate the percentage of NMAs for which evidence against the hypothesis of consistency is evident. We updated our previous empirical evaluation using a larger sample of published NMAs with dichotomous outcome data [10]. We also aimed to describe the association between evidence against consistency and the NMA structural characteristics, such as number of studies, interventions, and loops. We finally aimed to evaluate the amount of heterogeneity in consistency and inconsistency models.

## Methods

### Eligibility criteria for network database

The collection of published NMAs used in this paper has been described elsewhere [1, 2] (see also Additional file 1: Appendix 1). We included networks published in two distinct periods: 1) up to December 2015 (including NMAs identified from our previous search up to April 2015 [18], and from our updated search up to December 2015), and 2) between 2017 and 2018 (from our updated search up to July 2018 [19]). NMAs were eligible if they included only randomised clinical trials, had at least four intervention nodes (including placebo) in the network, had conducted any form of valid indirect comparison or NMA, included at least one loop, and had a dichotomous primary outcome with available arm-level data. Each eligible article contributed only one network, including the primary outcome, as reported in the publication or, if this was unclear, the first outcome conducting a NMA presented in the article.

### Synthesis

We performed a descriptive analysis of the eligible networks regarding the following characteristics: number of included studies, interventions, comparisons with direct evidence, presence of at least one intervention comparison informed by a single study, multi-arm studies, loops, number of unique designs, presence of complex interventions (as defined by Welton et al. [20]), type of outcome (objective, semi-objective and subjective), and type of intervention comparisons (pharmacological versus pharmacological, pharmacological versus placebo, non-pharmacological versus any) [2, 21]. In particular, NMAs including pharmacological interventions and a placebo or control were categorized in the pharmacological versus placebo comparison type. NMAs with only pharmacological interventions were categorized in the pharmacological vs pharmacological comparison type, whereas NMAs with at least one non-pharmacological intervention were categorized in the non-pharmacological vs any intervention comparison type.

We assessed consistency in each network using the DBT model that evaluates the entire network as a whole and encompasses the potential conflict between studies including different sets of interventions, named ‘designs’ [6, 8]. In this model we synthesised evidence in a way that reflects the extra variability due to inconsistency (i.e., beyond what is expected by heterogeneity or random error) [22], and encompassed the potential conflict between studies with different sets of interventions [6, 8]. We assessed evidence against the hypothesis of consistency based on the *p*-value of the DBT test (see Additional file 1: Appendix 2). Since the tests of inconsistency are known to have low power [17, 23], and considering that empirical evidence showed that 10% of loops are inconsistent at *p* < 0.05 [10], we decided to use along with the commonly used cut-off *p*-value of 0.05, the cut-off *p*-value of 0.10.

We estimated the prevalence of NMAs for which there was evidence or strong evidence against the hypothesis of consistency was evident (at both 0.10 and 0.05 thresholds) and explored its association with network structural characteristics. We present scatterplots and box plots for the aforementioned descriptive characteristics against the *p*-value of the DBT test. We visually assessed if inconsistency rate changed per year of study publication in a stacked bar plot. To explore the association between the DBT *p*-value and prevalence of evidence of inconsistency with estimation of heterogeneity in consistency and inconsistency models, we plotted the estimated between-study standard deviation values under the consistency and inconsistency models. We used a different colour scheme for each network to indicate evidence against the consistency hypothesis at 0.05 and 0.10 thresholds. To evaluate jointly the influence of the number of studies, interventions, direct intervention comparisons, and loops on the DBT *p*-value, we performed a multivariable regression analysis of the *p*-value with the *glm* function in R [24]. We also estimated the Pearson correlation coefficients to capture any dependencies between the *p*-values of the DBT model against the network structural characteristics, using a logarithmic scale.

Among the several approaches that have been suggested to estimate the between-study variance we selected the popular DerSimonian and Laird (DL) method and the restricted maximum likelihood (REML) method which has been shown to be a better alternative [25, 26]. For completeness, we investigated the impact of both ways to estimate the between-study variance on the consistency evaluation. We note that estimated heterogeneity in the inconsistency models represents within-design heterogeneity only, while estimated heterogeneity in the consistency models represents both within- and between-design heterogeneity (Additional file 1: Appendix 2). We conducted both consistency and inconsistency models in Stata and R using the *network* [27] suite of commands and *netmeta* [28] package, respectively. We used both Stata and R software, since at the time of conducting the analyses the REML estimator for heterogeneity was available in the *network* [27] command in Stata only, and the DL estimator in the *netmeta* [28] R package. Currently, both REML and DL estimators for heterogeneity are available in the *netmeta* [28] package. We also calculated the I-squared statistic for each network using the *netmeta* package in R and assessed its association with the DBT *p*-values in a scatterplot.

## Results

### Description of the network database

From the 456 total NMAs identified from our previous search [2], we located 105 NMAs satisfying the eligibility criteria. Using the same process for the years 2015 (April 2015 to December 2015), 2017 and 2018 we also included another 96 NMAs. Overall, we included and analysed 201 NMAs that fulfilled the eligibility criteria, corresponding to 201 networks (Additional file 1: Appendix Figure 1).

The median number of studies per network was 20 (IQR 13, 35), and the median number of interventions per network was seven (IQR 5, 9). Multi-arm trials were included in 142 networks (70%), with a median number of multi-arm studies one (IQR 0, 3). The median number of the unique direct intervention comparisons in the included networks was 10 (IQR 6, 14), whereas the median number of unique designs was eight (IQR 6, 13). Most networks included at least one comparison (186 networks, 92.5%) informed by a single study. The median number of loops across networks was three (IQR 2, 7), and the median number of inconsistency parameters per network was four (IQR 2, 7) (see also Additional file 1: Appendix 1). The median I-squared statistic was 30% (IQR 0, 59%).

### Prevalence of inconsistency

At the 0.05 threshold, evidence against the consistency hypothesis was detected in 28 (14%) networks when using REML. At the 0.10 level, strong evidence against the consistency hypothesis was detected in 39 (20%) networks of the 201 total networks with REML. Changing from REML to DL estimators for heterogeneity had only a minor impact on the prevalence of evidence of inconsistency (see Additional file 1: Appendix Table 1) [25]. Most DBT *p*-values were considerably higher than 0.10, irrespective of heterogeneity estimator. No change in the prevalence of evidence of inconsistency was detected across years (*p*- value = 0.39, Additional file 1: Appendix Figure 2).

In the following, results are presented at the 0.05 threshold and according to the REML estimation method for the within design heterogeneity variance of overall heterogeneity variance.. Results according to the DL estimator are presented in the supplementary files.

#### Evidence of inconsistency across different network structural characteristics

**Fig. 1:**
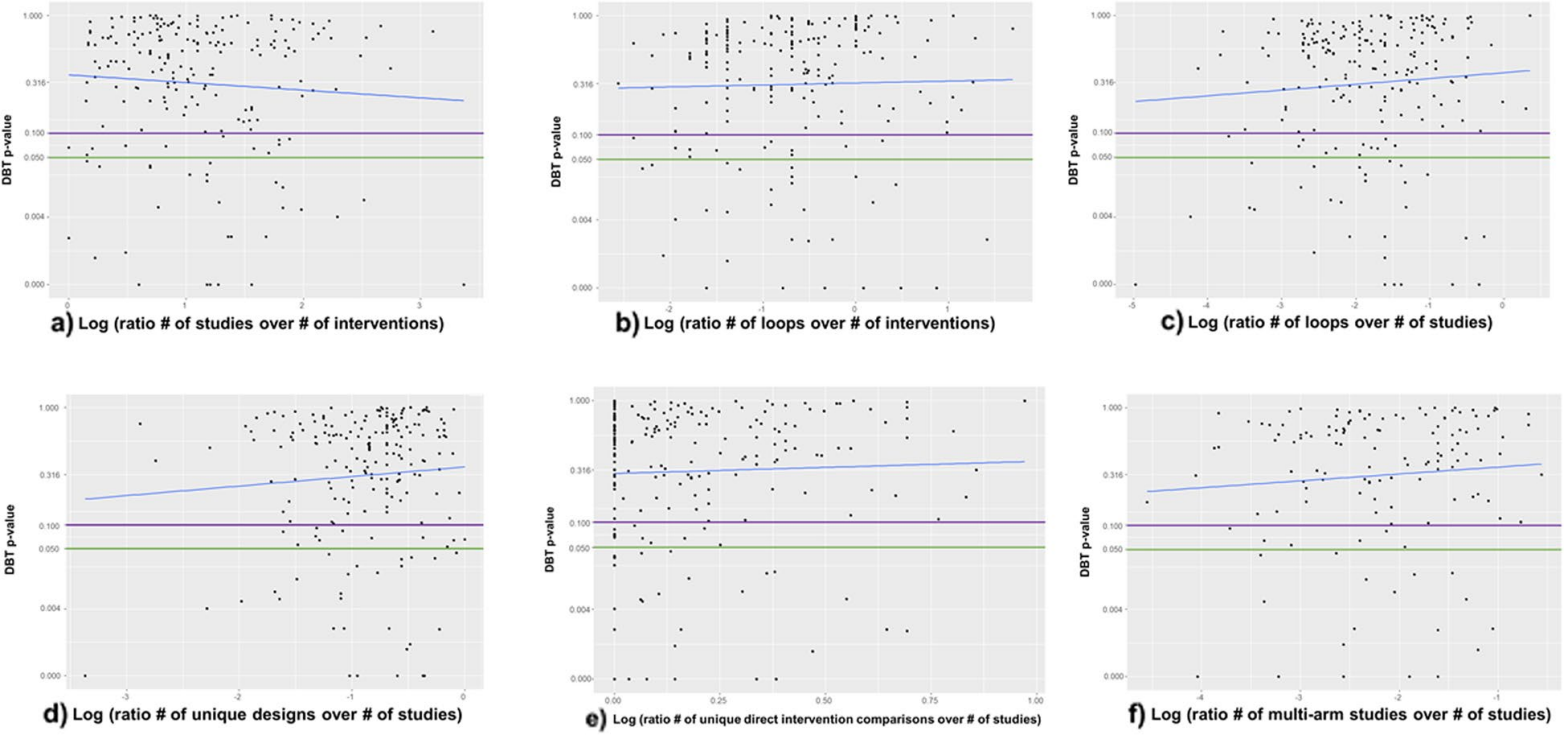
Plot of *p*-values (fourth-root scale) of the DBT model against ratios of network structural characteristics (logarithmic scale). **a** Explores the ratio of the number of studies to the number of interventions in a network (correlation coefficient [*p*-value] = − 0.07 [0.31]); **b** Explores the ratio of the number of loops to the number of interventions in a network (correlation coefficient [*p*-value] = 0.02 [0.72]); **c** Explores the ratio of the number of loops to the number of studies in a network (correlation coefficient [*p*-value] = 0.08 [0.28]); **d** Explores the ratio of the number of unique designs to the number of studies in a network (correlation coefficient [*p*-value] = 0.07 [0.28]); **e** Explores the ratio of the number of unique direct intervention comparisons to the number of studies in a network (correlation coefficient [*p*-value] = 0.06 [0.40]); **f** Explores the ratio of the number of multi-arm studies to the number of total studies in a network (correlation coefficient [*p*-value] = 0.09(0.19)). All analyses have used the REML estimator for heterogeneity. The horizontal green and purple lines represent the cut-off *p*-value = 0.05 and *p*-value = 0.10, respectively. The blue diagonal line is the regression line. * Networks with no multi-arm studies were treated as networks including a single multi-arm study, to avoid excluding them from the plot, since the logarithm could not be calculated. Abbreviations: DBT, design-by-treatment interaction model; REML, restricted maximum likelihood

Lower *p*-values in the DBT test were more likely in networks with many studies, many direct intervention comparisons, and many designs (Additional file 1: Appendix Figure 3). However, these associations were rather weak (studies in the network: correlation coefficient = − 0.08 [*p*-value = 0.25], direct intervention comparisons in the network: correlation coefficient = − 0.07 [*p*-value = 0.33], designs in the network: correlation coefficient = − 0.03 [*p*-value = 0.67]) and Fig. 1 shows that most factors do not greatly influence the p-value of inconsistency. A weak association was found between the ratio of the number of studies to the number of interventions the *p*-values in DBT (correlation coefficient = − 0.07 [*p*-value = 0.31]; see also Additional file 1: Appendix Figure 4 for results using the DL heterogeneity estimator). This was expected as power in detecting inconsistency is higher in networks with many studies per intervention comparison. It should also be noted that networks with few studies and many interventions were associated with larger heterogeneity, which can mask detection of inconsistency (Additional file 1: Appendix Figure 5). Overall, the correlation analyses did not reveal an important association between network characteristics and DBT test *p*-values.

In line with the weak associations found in Fig. 1, the multivariable regression analysis showed that none of the number of included studies in the network, number of interventions, number of unique pairwise comparisons and number of loops affected significantly the DBT *p*-values (Additional file 1: Appendix Table 2).

**Fig. 2:**
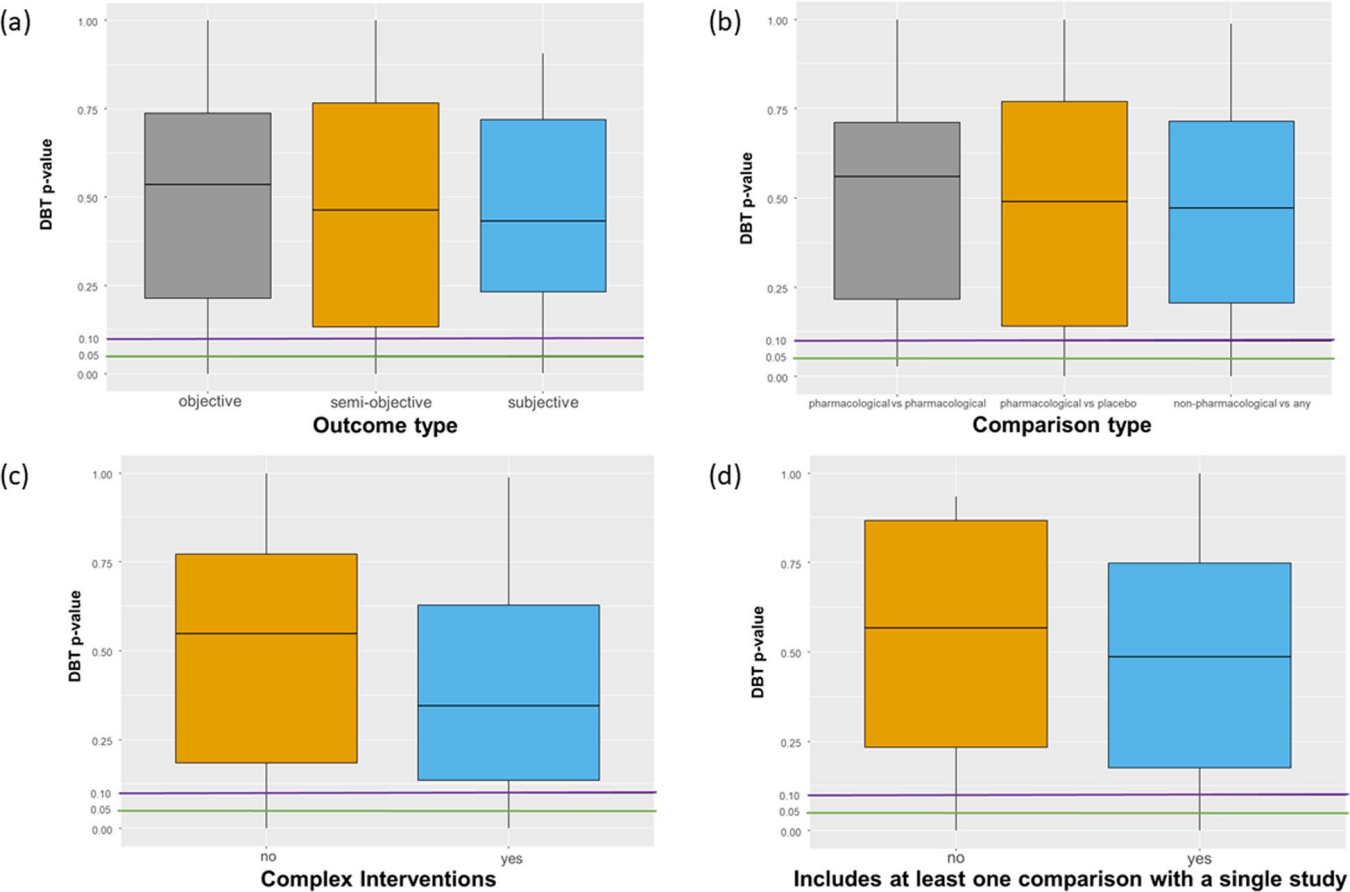
Box plot of *p*-values (fourth-root scale) of the DBT model per **a** type of outcome, **b** type of intervention comparison, **c** presence of at least one direct intervention comparison with a single study, and **d** presence of complex interventions. The horizontal green and purple lines represent the cut-off *p*-value = 0.05 and *p*-value = 0.10, respectively. All analyses have used the REML estimator for heterogeneity. Abbreviations: DBT, design-by-treatment interaction model; REML, restricted maximum likelihood

The type of outcome (*p*-value = 0.86), type of intervention comparisons in the network (*p*-value = 0.75), and the presence of complex interventions (*p*-value = 0.08) did not suggest important differences in the distribution of the *p*-values calculated in DBT. Similarly, the inclusion of at least one intervention comparison with a single study in the network did not affect the assessment of the global inconsistency in the network (*p*-value = 0.57) (Fig. 2 and Additional file 1: Appendix Figure 6).

### Heterogeneity in consistency and inconsistency models

**Fig. 3:**
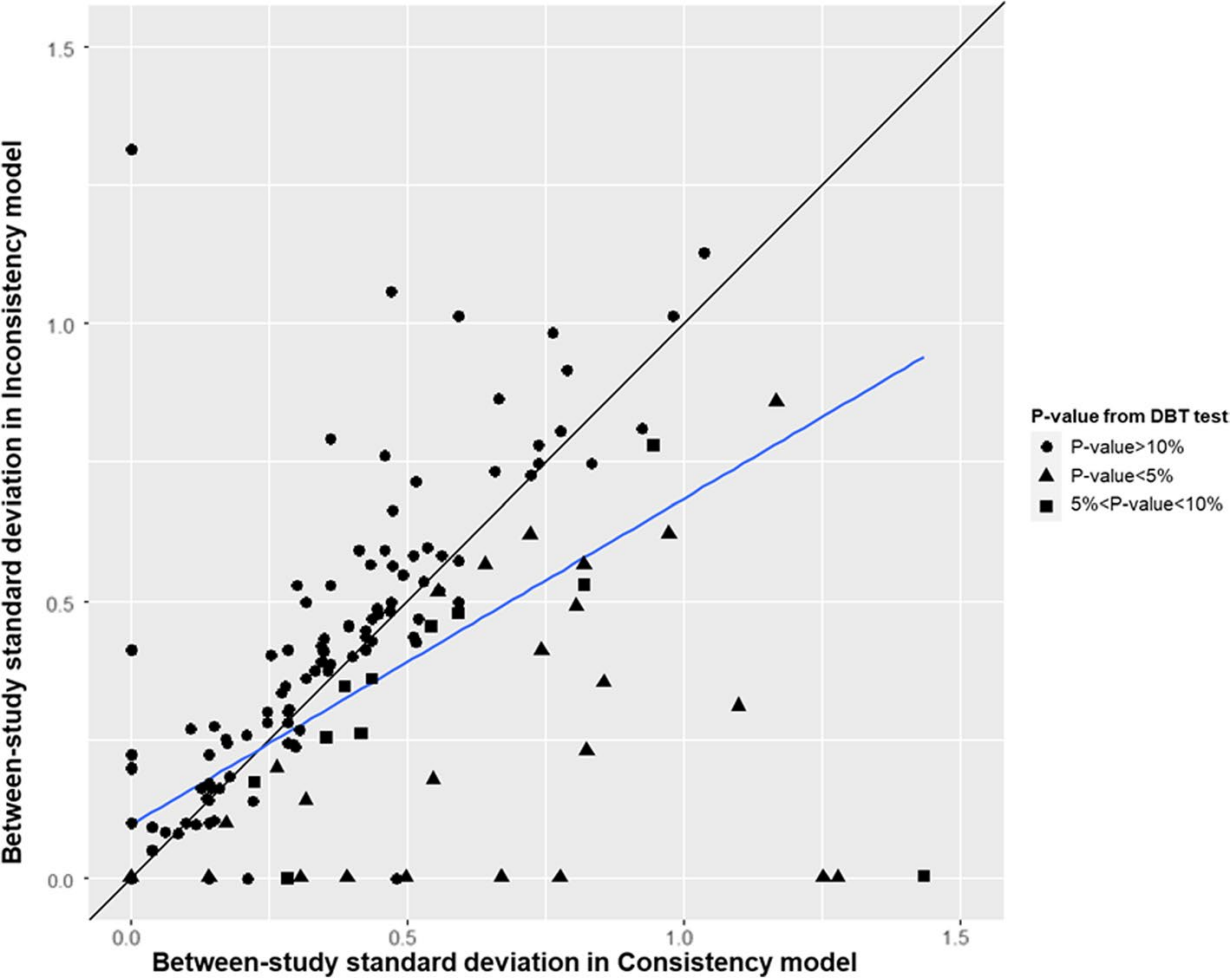
Plot of the between-study standard deviation in consistency against the inconsistency model. The black diagonal line represents equality in between-study standard deviation between consistency and inconsistency models. Circle points represent the networks consistent at the threshold of 10%, triangular points represent the inconsistent networks at α = 5%, and rectangular points represent networks inconsistent between the thresholds 5 and 10%. All analyses have used the REML estimator for heterogeneity. Abbreviations: DBT, design-by-treatment interaction model; REML, restricted maximum likelihood

An increase in the I-squared statistic was associated with a decrease in the DBT *p*-value (Additional file 1: Appendix Figure 7). In Fig. 3, we plot the estimated between-study standard deviation values under the consistency and inconsistency models by levels of evidence against the consistency hypothesis (see also Additional file 1: Appendix Figure 8 for results using the DL heterogeneity estimator). Evidence against the consistency assumption was associated with heterogeneity being larger in the consistency model compared to the inconsistency model. In some networks the within-design heterogeneity (estimated in the inconsistency model) is very small or zero, but the number of the degrees of freedom of the Wald chi-squared test is large with evidence of inconsistency (Additional file 1: Appendix Figure 9). Higher overall heterogeneity (estimated in the consistency model) when compared with zero within-design heterogeneity suggests evidence of inconsistency, and this is driven by between-design heterogeneity. For zero within-design heterogeneity, we found 13 networks with the DL estimator and 15 networks with the REML estimator that estimate higher overall heterogeneity.

## Discussion

Our findings suggest that evidence of inconsistency was at least twice as frequent as what would be expected by chance if all networks were truly consistent (when we would expect one in 20 networks for 0.05 level, and one in 10 networks for 0.10 level under the null hypothesis of no inconsistent networks in our sample). Overall, evidence against the hypothesis of consistency (as defined by the *p*-value of the DBT test) in NMAs with dichotomous data was evident in one in seven networks using the threshold of 0.05. Taking into consideration the low power of the inconsistency test, and in particular the DBT model that has more degrees of freedom in contrast to other inconsistency tests [16, 17], we decided to use also the threshold of 0.10, where inconsistency was prevalent in one in five networks. Considering that the observed inconsistent NMAs are at 14% of the networks, we expect that the truly inconsistent networks range between 12 and 20%, assuming the test has a perfect type I error at 0.05 and power ranges between 50 and 80%.

Our study showed that structural network characteristics only weakly impact the detection of inconsistency, potentially due to lack of powering of testing inconsistency in most of the included networks. In particular, we found a mild association between networks including both a high number of studies and a small number of interventions or loops, and lower *p*-values of the DBT test. This is probably due to the increased power to detect inconsistency in these types of networks. Another key finding of our study was that an important drop in heterogeneity when moving from the consistency to the inconsistency model is associated with evidence of inconsistency. This suggests that heterogeneity estimated in the consistency model may account for discrepancies between direct and indirect evidence in the network. A sharp rise in overall heterogeneity as compared to the within-design heterogeneity in case of large inconsistency, is also explained through the definition of heterogeneity parameters in the consistency (overall heterogeneity) and inconsistency models (within-design heterogeneity) (see also Additional file 1: Appendix 2). Results were overall consistent among DL and REML heterogeneity estimators.

To the best of our knowledge, this is the largest empirical study used to evaluate the prevalence of evidence of inconsistency in networks of trial evidence. Overall, our findings are aligned with our previous study [10], where we evaluated 40 networks of interventions. The present research study includes five times the number of networks included in our previous review, and the exploration of multiple structural network characteristics. In this study, we found equal to empirical rates of inconsistency compared with our previous study (i.e., 14% of 201 networks vs. 13% of the 40 networks), suggesting that researchers should devote more resources to exploring how to mitigate inconsistency.

Our study has a few limitations worth noting. First, for the empirical assessment of consistency, we evaluated articles with dichotomous outcome data restricting to the odds ratio effect measure. We expect our findings to be generalisable to other effect measures. Although our previous empirical study showed that in some cases inconsistency was reduced when moving from one effect measure to another, overall, the detected inconsistency rates were similar for different effect measures [10]. For completeness it would be interesting to carry out an empirical study for continuous outcomes to examine possible differences in inconsistency between mean differences, standardized mean differences and ratios of means. Second, in the present study we considered a common within-network heterogeneity. This is often clinically reasonable and statistically convenient. Since most direct intervention comparisons in networks comprise only few studies, sharing the same amount of heterogeneity allows such comparisons to borrow strength from the entire network. However, assuming common within-network heterogeneity, intervention comparisons with a smaller heterogeneity than that of the remaining network will be associated with a larger reported uncertainty around their summary effect, compared to what would be accurate. In such a case, the chances of detecting inconsistency decrease. Although assuming a common within-network heterogeneity can underestimate inconsistency, it better reflects how summary effects are combined in an NMA in practice. Alternatively, when heterogeneity is believed to vary across comparisons, different heterogeneity parameters can be built into the model, but need to be restricted to conform to special relationships according to the consistency assumption [29]. Third, we assessed detection of inconsistency based on a threshold of the DBT *p*-value, which reflects common practice, and ignored the actual differences between the different designs and the direct and indirect estimates. However, to avoid “vote-counting” of evidence against the consistency hypothesis we also explored the distribution of the DBT *p*-values according to several network structural characteristics. Fourth, we used the Wald test statistic, which under consistency follows a χ^2^ distribution, but like any global test it may lack power. Therefore, when the hypothesis of consistency is not rejected, inconsistency may be present, and one should also consider the nature and design of the included studies before making any inferences. However, our results rely on the assumption that the Wald test statistic has a chi-squared distribution under the null. A reviewer has suggested that this assumption is not always valid. In particular, the validity of the Wald chi-square test improves as the degrees of freedom of the test increase and the within-design heterogeneity variance decreases. This suggests that false positive inconsistency results (i.e., low DBT *p*-values) may be apparent with small degrees of freedom and large within-design heterogeneity, whereas true positive inconsistency may result from large degrees of freedom and small within-design heterogeneity (see Additional file 1: Appendix Figure 9 for a plot of the between-study standard deviation in inconsistency against the degrees of freedom of the Wald chi-square test). Fifth, we did not exclude potential outlier networks, since this was outside of the scope of the study. Sixth, our investigation of inconsistency relies on what the original authors included into their NMAs, and lower inconsistency could have been identified if we restricted our analysis to high-quality systematic reviews and NMAs. However, it should be noted that inconsistency, similar to between-study heterogeneity, is not a matter of study quality per se, but it should (or not) be expected.

In a systematic review and NMA, investigators should interpret evidence against the consistency hypothesis very carefully and be aware that inconsistency in a network can be absorbed into estimates of heterogeneity. Given that the descriptive prevalence of evidence of inconsistency is frequent in published NMAs, authors should be more careful in the interpretation of their results. Confidence in the findings from NMA should always be evaluated, using for example the tools Confidence In Network Meta-Analysis (CINeMA [30]) or Grading of Recommendations Assessment, Development, and Evaluation (GRADE) for NMA approaches [31, 32]. Since inconsistency tests may lack power to identify true inconsistency, we recommend avoiding interpreting ‘no evidence for inconsistency’ as ‘no inconsistency’. We also recommend using both a global (e.g., the DBT model) and a local approach (e.g., loop-specific approach [10] or node-splitting [7] method) for the assessment of inconsistency in a network, before concluding about the absence or presence of inconsistency. However, detection of inconsistency often prompts authors to choose only direct evidence, which is often perceived as less prone to bias, disregarding the indirect information [23]. It is advisable though, instead of selecting between the two sources of evidence, to try to understand and explore possible sources of inconsistency and refrain from publishing results based on inconsistent evidence [5, 33].

NMA is increasingly conducted and although assessment of the required assumptions has improved in recent years, there is room for further improvement [1, 2]. Systematic reviews and NMA protocols should present methods for the evaluation of inconsistency and define strategies to be followed when inconsistency is present. The studies involved in an NMA should also be compared with respect to the distribution of effect modifiers across intervention comparisons. Authors should follow the PRISMA (Preferred Reporting Items for Systematic Review and Meta-analysis)-NMA guidelines [34] and report their inconsistency assessment results, as well as the potential impact of inconsistency in their NMA findings.

Given that inconsistency is frequent in nature (with up to 20% of networks expected to be inconsistent), investigators should be more careful in the interpretation of their results. Our results highlight the need for a widespread use of tools that assess confidence in the NMA findings, such as CINeMA [30], that factor-in inconsistency concerns in the interpretation. Also, our findings shed more light on the drivers of power for the consistency test, and underline that it is essential to develop strategies to detect inconsistency, particularly in cases where the existing tests have low power. Considering that the power for detecting inconsistency is low in many networks, it is difficult to judge whether inconsistency is rare or it is common but commonly not detectable. *P*-values of the global Wald test should be interpreted with caution since, similar to all global tests, they may lack power, as well as they are calculated under the assumption that the within-design heterogeneity is known. In particular, in networks with only a few degrees of freedom available for estimating the within-design heterogeneity parameter, this assumption is most likely not justified. More investigation is also needed to evaluate the performance of other inconsistency methods (e.g., node-splitting [7], Lu and Ades method [12]) and to understand their power under different meta-analytical scenarios. Further investment is required in developing methods to potentially deal with inconsistent networks (e.g., network meta-regression approaches, NMA methods for classes of interventions) and to educate researchers about their use. Careful considerations should also be made when building the network geometry and when deciding what to include in a network (e.g., different doses and formulations). Another key consideration to help reduce inconsistency is to include studies relevant to the research question, where study populations, interventions, outcomes, and study settings can be representative of the settings, populations, and outcomes of the systematic review (i.e., see indirectness in CINeMA [30] and GRADE [32]).”

## Conclusion

Evidence of inconsistency is more frequent than what would be expected by chance if all networks were consistent. This suggests that inconsistency should be appropriately explored. This empirical study shows that detection of inconsistency was mildly sensitive to various network characteristics and their combination. Networks with a high number of studies, and a small number of interventions had larger power to detect inconsistency. Also, inconsistency was likely to manifest as extra heterogeneity when the consistency model was fitted. Lower estimates of heterogeneity in the inconsistency model compared with the consistency model were associated with higher rates of detection of inconsistency. Overall, there was a good empirical agreement of inconsistency when different heterogeneity estimation methods were used.

## Supplementary Information

**Additional file 1: Appendix 1.** Eligibility criteria, screening, study selection, and data abstraction. **Appendix 2.** Model description. **Appendix Figure 1.** Flowchart for network meta-analysis study inclusion. **Appendix Table 1.** Number of consistent and inconsistent networks at 0.05 and 0.10 significance levels using DL and REML heterogeneity estimators. **Appendix Table 2.** Multivariable regression analysis results. **Appendix Figure 2.** Stacked bar plot of consistent (green bars) and inconsistent (blue bars) networks at α = 0.05 per heterogeneity estimator and year of study publication^‡^. **Appendix Figure 3.** Plot of *p*-values (fourth-root scale) of the DBT model against network structural characteristics (logarithmic scale). **Appendix Figure 4.** Plot of *p*-values (fourth-root scale) of the DBT model against ratios of network structural characteristics (logarithmic scale). **Appendix Figure 5.** Plot of the between-study standard deviation estimated in the consistency model against the ratio of the number of studies to the number of interventions in a network (logarithmic scale). **Appendix Figure 6.** Box plot of the *p*-values (fourth-root scale) of the DBT model per (a) type of outcome, (b) type of intervention comparison, c) presence of complex interventions, and d) presence of at least one direct intervention comparison with a single study. **Appendix Figure 7.** Plot of the *p*-values (fourth-root scale) of the DBT model against the I-squared. **Appendix Figure 8.** Plot of the between-study standard deviation in consistency against the inconsistency model. **Appendix Figure 9.** Plot of the between-study standard deviation in inconsistency against the degrees of freedom of the Wald chi-square test (logarithmic scale).

## Data Availability

The datasets used and/or analysed during the current study are available through the *nmadb* R package (Network Meta-Analysis Database API; https://cran.r-project.org/web/packages/nmadb/index.html).

## Abbreviations

DBT: Design-by-treatment
DL: DerSimonian and Laird
IQR: Interquartile Range
NMA: Network meta-analysis
REML: Restricted maximum likelihood
