# Supplementary material for: Prevalence of evidence of inconsistency and its association with network structural characteristics in 201 published networks of interventions
Source: BMC Med Res Methodol. 2021 Oct 25;21:224. doi: 10.1186/s12874-021-01401-y (PMC8543923; doi:10.1186/s12874-021-01401-y)

Supplementary Online Content

Appendix 1. Eligibility criteria, screening, study selection, and data abstraction 2

Appendix 2: Model description 4

Appendix Figure 1: Flowchart for network meta-analysis study inclusion 8

Appendix Table 1: Number of consistent and inconsistent networks at 0.05 and 0.10 significance levels using DL and REML heterogeneity estimators. 9

Appendix Table 2: Multivariable regression analysis results. 9

Appendix Figure 2: Stacked bar plot of consistent (green bars) and inconsistent (blue bars) 10

networks at α=0.05 per heterogeneity estimator and year of study publication‡ 10

Appendix Figure 3: Plot of p-values (fourth-root scale) of the DBT model against network structural characteristics (logarithmic scale) 11

Appendix Figure 4: Plot of p-values (fourth-root scale) of the DBT model against ratios of network structural characteristics (logarithmic scale) 15

Appendix Figure 5: Plot of the between-study standard deviation estimated in the consistency model against the ratio of the number of studies to the number of interventions in a network (logarithmic scale) 17

Appendix Figure 6: Box plot of the p-values (fourth-root scale) of the DBT model per (a) type of outcome, (b) type of intervention comparison, c) presence of complex interventions, and d) presence of at least one direct intervention comparison with a single study 18

Appendix Figure 7: Plot of the p-values (fourth-root scale) of the DBT model against the I-squared 19

Appendix Figure 8: Plot of the between-study standard deviation in consistency against the inconsistency model 20

Appendix Figure 9: Plot of the between-study standard deviation in inconsistency against the degrees of freedom of the Wald chi-square test (logarithmic scale) 21

Appendix 1. Eligibility criteria, screening, study selection, and data abstraction

An expert librarian developed the literature search for MEDLINE, EMBASE and the Cochrane Database of Systematic Reviews to identify network meta-analyses (NMAs) of randomized controlled trials (RCTs). We searched from inception until July, 2018. A second librarian peer-reviewed the search using the Peer Review of Electronic Search Strategies (PRESS) checklist[1]. The final search strategy for the MEDLINE database is available elsewhere[2-4].

We included NMAs that compared at least four interventions, including different drugs, medical treatments, schedules, doses or formulations of the same treatment, plus placebo, no treatment, waiting list or control. NMAs written in English with a valid statistical method for indirect comparisons (e.g., adjusted indirect comparison method [5]) or NMAs (e.g., hierarchical models) were included.

We excluded publications with unadjusted indirect comparisons,[6] diagnostic test accuracy NMAs, and NMAs of non-randomized studies. We also excluded NMAs conducted in animals, as well as NMAs including a lower number of trials than the number of interventions compared. In the present study, we restricted to articles published in two distinct periods: 1) up to December 2015 (including NMAs identified from our previous search up to April 2015[7], and from our updated search up to December 2015), and 2) between 2017 and 2018 (from our updated search up to July 2018[8]). Given that this study received no funding, we decided to set up a threshold of the total included NMAs in this study, which was approximately 200 networks. Each eligible article contributed only one network.

We conducted a pilot-test of the eligibility criteria on a random sample of 30 articles prior to embarking the full screening process. Titles and abstracts were screened by two reviewers, independently (level 1). Included publications in level 1 were assessed in full-text for relevance by two reviewers (level 2). We used the online tool Synthesi.SR for the screening process.[9] Conflicts were resolved by discussion or the inclusion of a third reviewer.

For each included NMA we extracted the trial data of the primary/main outcome (as reported in the publication or, if this was unclear, defined as the first outcome presented). We abstracted data presented in 2×2 tables, including the name of each trial (i.e. author name and year of publication, where available), the number of events, the sample size and the arm-specific intervention of each trial included in the NMA. Data abstraction was performed by a single reviewer.

References

1. McGowan J, Sampson M, Salzwedel DM, Cogo E, Foerster V, Lefebvre C: **PRESS Peer Review of Electronic Search Strategies: 2015 Guideline Statement**. *Journal of clinical epidemiology* 2016, **75**:40-46.
2. Petropoulou M, Nikolakopoulou A, Veroniki AA, Rios P, Vafaei A, Zarin W, Giannatsi M, Sullivan S, Tricco AC, Chaimani A *et al*: **Bibliographic study showed improving statistical**

methodology of network meta-analyses published between 1999 and 2015. *Journal of clinical epidemiology* 2017, **82**:20-28.
3. Zarin W, Veroniki AA, Nincic V, Vafaei A, Reynen E, Motiwala SS, Antony J, Sullivan SM, Rios P, Daly C *et al*: **Characteristics and knowledge synthesis approach for 456 network meta-analyses: a scoping review**. *BMC medicine* 2017, **15**(1):3.
4. Nikolakopoulou A, Chaimani A, Veroniki AA, Vasiliadis HS, Schmid CH, Salanti G: **Characteristics of networks of interventions: a description of a database of 186 published networks**. *PloS one* 2014, **9**(1):e86754.
5. Bucher HC, Guyatt GH, Griffith LE, Walter SD: **The results of direct and indirect treatment comparisons in meta-analysis of randomized controlled trials**. *J Clin Epidemiol* 1997, **50**(6):683-691.
6. Song F, Loke YK, Walsh T, Glenny AM, Eastwood AJ, Altman DG: **Methodological problems in the use of indirect comparisons for evaluating healthcare interventions: survey of published systematic reviews**. *BMJ* 2009, **338**:b1147.
7. Papakonstantinou T: **nmadb: Network Meta-Analysis Database API**. *https://CRANRprojectorg/package=nmadb* 2019.
8. Veroniki A.A., Tsokani S., Zevgiti S., Pagkalidou I., Kontouli K.M., Ambarcioglu P., Pandis N., Lunny C., Nikolakopoulou A., Papakonstantinou T. *et al*: *Systematic Reviews* 2021.
9. Knowledge Translation Program: **Synthesi.SR.** In. Toronto, Ontario: Li Ka Shing Knowledge Institute, St. Michael's Hospital; 2014.
10. White IR: **Network meta-analysis**. *Stata Journal* 2015, **15**:1-34.
11. Higgins JP, Jackson D, Barrett JK, Lu G, Ades AE, White IR: **Consistency and inconsistency in network meta-analysis: concepts and models for multi-arm studies**. *Res Synth Methods* 2012, **3**(2):98-110.
12. White IR, Barrett JK, Jackson D, Higgins JPT: **Consistency and inconsistency in network meta-analysis: model estimation using multivariate meta-regression**. *Res Synth Methods* 2012, **3**(2):111-125.
13. Jackson D, Barrett JK, Rice S, White IR, Higgins JPT: **A design-by-treatment interaction model for network meta-analysis with random inconsistency effects**. *Stat Med* 2014, **33**(21):3639-3654.
14. Krahn U, Binder H, Konig J: **A graphical tool for locating inconsistency in network meta-analyses**. *BMC Med Res Methodol* 2013, **13**:35.
15. Lu G, Welton NJ, Higgins JPT, White IR, Ades AE: **Linear inference for mixed treatment comparison meta-analysis: a two-stage approach**. *Res Synth Methods* 2012, **3**(3):255.
16. Veroniki AA, Vasiliadis HS, Higgins JPT, Salanti G: **Evaluation of inconsistency in networks of interventions**. *Int J Epidemiol* 2013, **42**(1):332-345.
17. Hutton B, Salanti G, Caldwell DM, Chaimani A, Schmid CH, Cameron C, Ioannidis JP, Straus S, Thorlund K, Jansen JP *et al*: **The PRISMA extension statement for reporting of systematic reviews incorporating network meta-analyses of health care interventions: checklist and explanations**. *Ann Intern Med* 2015, **162**(11):777-784.

Appendix 2: Model description

Notation

Consider a network of evidence comprising S interventions forming the set $\Omega =$ and N studies in total. Each study $i = 1, \dots, N$ compares a specific number of interventions. Let A be an arbitrarily chosen reference intervention and M an index for any of the S-1 remaining interventions. We use y_i^{AM} to refer to an observed effect size (e.g., log odds-ratio [LOR]) in study *i*, with the descriptor 'AM' specifying that the estimate refers to a comparison of intervention M relative to intervention A. The underlying parameter μ^{AM} refers to the average relative intervention effect of M relative to A. Any parameter μ^{AM} that includes intervention A is named a *basic* parameter and all other parameters are named *functional.* Under the assumption of consistency, a functional parameter associated with intervention comparison MX can be expressed via the consistency equation $\mu^{\mathrm{MX}} = \mu^{\mathrm{AX}} - \mu^{\mathrm{AM}}$.

Consistency and inconsistency models

To describe the consistency and inconsistency models it is ideal, but not necessary, to assume that all studies include the reference intervention A. In such a case, if a study does not include an A arm we impute data with minimal information as described by White *et al.*[10] to derive the observed effect sizes. Alternatively, the contrast-based format can be used, where the observed effect size comparing two interventions is used directly.

In the random-effects consistency model the observed intervention effect y_i^{AM} is modelled as

$$y_i^{AM} = \mu^{\mathrm{AM}} + \delta_i^{\mathrm{AM}} + e_i^{\mathrm{AM}}$$

where δ_i^{AM} is the random effect for study *i,* reflecting heterogeneity between studies comparing M to A. The term e_i^{AM} is the within-study error with the vector of the within-study errors being normally distributed $\boldsymbol{e_i} \sim N(\mathbf{0}, \mathbf{V}_i)$, where $\mathbf{V}_i$ is the within-study variance-covariance matrix assumed to be known. Note that for a two-arm study *i,* the within-study variance-covariance matrix $\mathbf{V}_i$ reduces to a scalar expressing the sample variance of the estimated intervention effect in study *i*. The study-specific random-effects are normally distributed with $\mathbf{T}$ denoting the between studies variance-covariance matrix

$$\boldsymbol{\delta_i} \sim N\left(\mathbf{0}, \mathbf{T} = \begin{pmatrix} \tau^2 & \dots & \tau^2/2 \\ \vdots & \ddots & \vdots \\ \tau^2/2 & \dots & \tau^2 \end{pmatrix}\right)$$

If the ith study is a two-arm study then the between studies variance-covariance matrix reduces to a scalar τ^2 expressing the between study variance. In the general case that a study compares K_i interventions, the dimension of both variance covariance matrices $\mathbf{T}$ and $\mathbf{V}_i$ is $(K_i - 1) \times (K_i - 1)$. When a network had no degrees of freedom for heterogeneity, the between-study variance could not be estimated, so we applied a common-effect DBT model (This happened when there was only one study for each design *d* in the network, see details below).

Let us assume that studies comparing the same subset of interventions belong to the same design *d*. Design *d* refers to studies with S_d specific interventions from the set Ω investigated in N_d studies and the network has in total *D* designs ($d = 1, \dots, D$). Consistency can conceptually fail to hold if there is difference between a direct (e.g., $\hat{\mu}^{\text{MX}}$) and an indirect (e.g., $\hat{\mu}^{\text{AX}} - \hat{\mu}^{\text{AM}}$) estimate for the same comparison or if there is difference in the relative effect of two interventions when it is estimated in studies with different designs.

Accounting for inconsistency, the DBT model for the observed intervention effect y_{id}^{AM}, with the descriptor *d* specifying the design of the study, is

$$y_{id}^{AM} = \mu^{\text{AM}} + \omega_d^{\text{AM}} + \delta_{id}^{\text{AM}} + e_{id}^{\text{AM}}$$

where the term ω_d^{AM} within design *d* represents inconsistency in the AM contrast, that is the amount of disagreement between different sources of evidence (variability in loops or between designs). The model accounts for the difference in estimating the relative effect AM when measured in different designs by adding the ω_d^{AM} term. If any AM comparison is estimated using direct evidence only (is not involved in a loop) and there are no multi-arm studies involving both A and M interventions then inconsistency cannot be estimated and we set $\omega_d^{\text{AM}} = 0$ to ensure identifiability. Also, ω_d^{AM} is ignored if design *d* does not contain A and M interventions. The number of inconsistency terms depends on both the total number of interventions in the network and the number of interventions in each design, and is obtained as $l = \sum_d (S_d - 1) - (S - 1)$ (see Higgins et *al[11]* and White et *al.*[12] for more details). Omission of the term ω_d^{AM} and

index *d* from the inconsistency model gives the consistency model. Therefore, l is the difference in the number of parameters between the inconsistency and consistency models.

The ω parameters might be treated either as random effects[13], assuming they all come from a common normal distribution $\omega_d^{\mathrm{AM}} \sim N(0, \sigma^2)$ with σ^2 the inconsistency variance, or as linearly independent fixed effects allowing different sources of evidence to differ by a fixed quantity[12]. In this paper we consider fixed ω parameters, and the DBT is applied in a frequentist setting. The model presentation considers the case when only two-arm trials are available. For the inclusion of multi-arm studies, extension is possible with some modification (e.g., in case three-arm trials are included, each yield two estimated treatment contrasts with within-study covariances assumed to be known).

To assess the presence of inconsistency in the entire network, we include all ω_d^{AM} inconsistency terms in a vector $\boldsymbol{\Omega}$ with dimension $l \times 1$ and assess the null hypothesis H_0: $\boldsymbol{\Omega} = \mathbf{0}$ using the following global Wald test statistic, which under a null consistency assumption follows a χ^2-test with *l* degrees of freedom:

$$\mathrm{W}^{DBT} = \boldsymbol{\Omega}'\boldsymbol{\Sigma}^{-1}\boldsymbol{\Omega}$$

where $\boldsymbol{\Sigma}$ is the $l \times l$ variance-covariance matrix of $\boldsymbol{\Omega}$. A p-value< 0.05 (or 0.10, depending on the cut-off decided a priori) supports rejecting the null of consistency. The model accounts for correlations across effect sizes estimated in multi-arm trials and is insensitive to their parameterisation.

Note that the W^{DBT} statistic under the common-effect model is equivalent to the Q-statistic for the evaluation of the assumption of consistency as presented elsewhere[14, 15]. In NMA, the assessment of homogeneity and consistency assumptions can be conducted using the generalized Cochran's Q-statistic for multivariate meta-analysis[14]. The Q-statistic in a common-effect model can be decomposed into the sum of the within-design Q-statistics and the between-designs Q-statistic. We calculated the Q and I-squared statistics for each network using the *netmeta* package in R. The *netmeta* package provides a single heterogeneity/inconsistency I-squared value from the Q statistic for the overall network based on the formula $\mathrm{I}^2 = \max\{0, 100\%(\mathrm{Q} - \mathrm{df})/\mathrm{Q}\}$.

We distinguish heterogeneity in the consistency and inconsistency models as: a) representing within- and between-design heterogeneity in the consistency model, and b) representing within-design heterogeneity only in the inconsistency model. This is because under the consistency assumption, the random-effects consistency model accounts for heterogeneity between studies. Between-study heterogeneity can be expressed as variability across studies within a design (e.g., across studies comparing interventions A vs. B) or as variability between designs (e.g., between designs A vs. B and A vs. B vs. C for studies comparing the common intervention comparison A vs. B). In case consistency fails to hold, the random-effects inconsistency model accounts for the difference in estimating a relative effect when measured in different designs by adding an extra term, the inconsistency factor. Through this factor the inconsistency model quantifies the extent of inconsistency in the network accounting for the variability coming between designs (e.g., variability between designs Avs.B and Avs.Bvs.C is expressed as design inconsistency). Hence, heterogeneity in the inconsistency model represents only within-design variability (e.g., variability across studies comparing interventions A vs. B).

Appendix Figure 1: Flowchart for network meta-analysis study inclusion

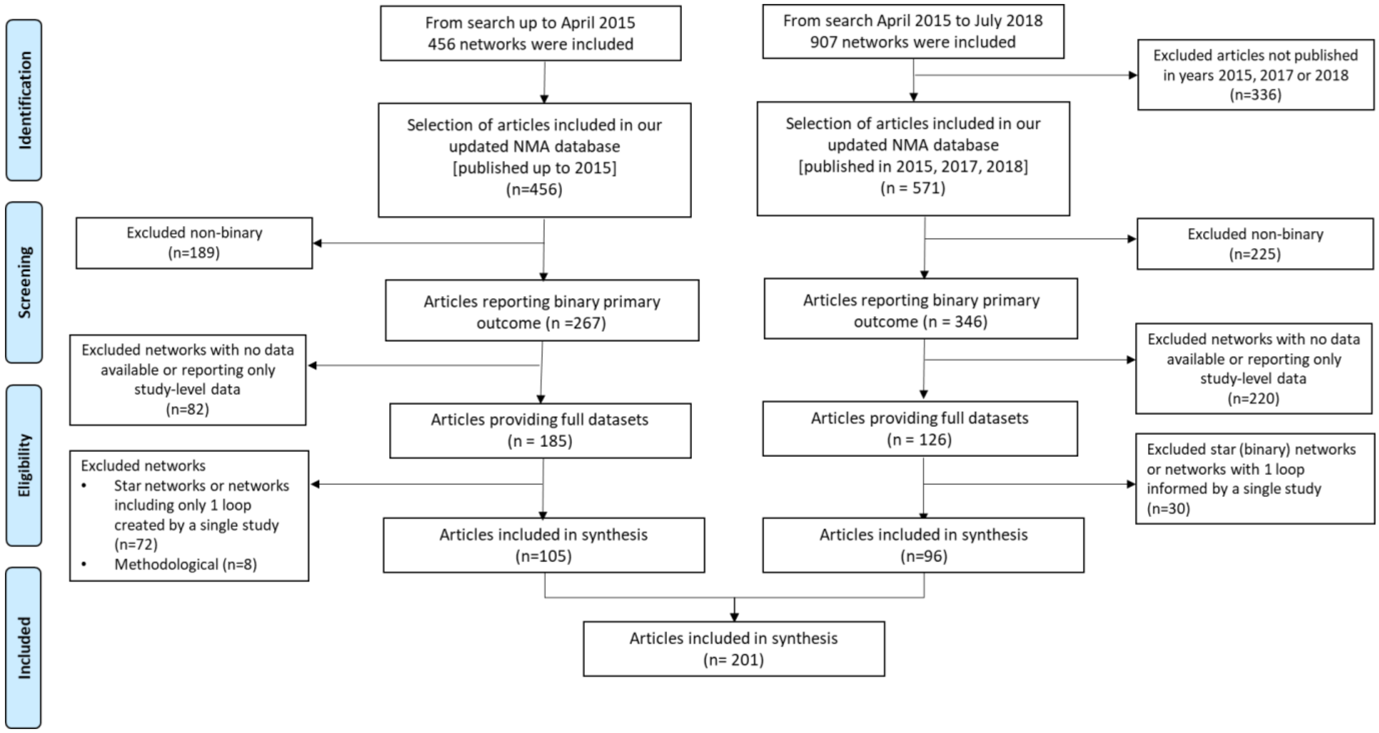

* Note: Considering that we located 105 NMAs published up to 2015, we decided to select the latest years from the updated search to include the most recently published NMAs. Hence, we included all identified NMAs with available data published in 2017 and 2018, and these were 96 NMAs. In total, we included 201 networks, which was in accordance with our initial aim (i.e., to include ~200 networks). Hence, we have now included 5 times more NMAs compared to our previous publication, where we had included 40 NMAs in total[16]. We believe that our decision to not include NMAs published in 2016 did not importantly change our results. Also, no important differences were found when the rates in the prevalence of evidence of inconsistency up to 2015 and after 2015 were compared (p-value= 0.869). It should also be noted that in June 2015 the PRISMA-NMA guidelines[17] were published, and this is a key chronological timepoint for NMAs.

Appendix Table 1: Number of consistent and inconsistent networks at 0.05 and 0.10 significance levels using DL and REML heterogeneity estimators.

Estimation Method	REML				Total # of Networks
		P<0.05	0.05≤P<0.10	P≥ 0.10	
DL	P<0.05	25 (12%)	3 (1.5%)	5 (2.5%)	**33 (16%)**
	0.05≤P<0.10	1 (0.5%)	7 (3.5%)	3 (1.5%)	**11 (6%)**
	P≥0.10	2 (1%)	1 (0.5%)	154 (77%)	**157 (78%)**
**Total # of Networks**		**28 (14%)**	**11 (6%)**	**162 (86%)**	**201 (100%)**

***** P is the p-value of the design-by-treatment inconsistency test
Abbreviations: DL, DerSimonian and Laird; REML, restricted maximum likelihood

Appendix Table 2: Multivariable regression analysis results.

		**DBT p-value***
**Heterogeneity Estimator**	**Network characteristics****	**Coefficient (95% CI)**
**REML**	# studies	-0.13 (-0.63, 0.35)
	# interventions	0.21 (-1.58, 2.01)
	# unique comparisons	-0.57 (-2.72, 1.53)
	# loops	0.38 (-0.35, 1.14)
**DL**	# studies	-0.23 (-0.73, 0.25)
	# interventions	0.07(-1.87, 1.74)
	# unique comparisons	-0.26 (-2.40, 1.85)
	# loops	0.27 (-0.46, 1.03)

*Logit transformed; **log transformed

Appendix Figure 2: Stacked bar plot of consistent (green bars) and inconsistent (blue bars) networks at α=0.05 per heterogeneity estimator and year of study publication[‡]

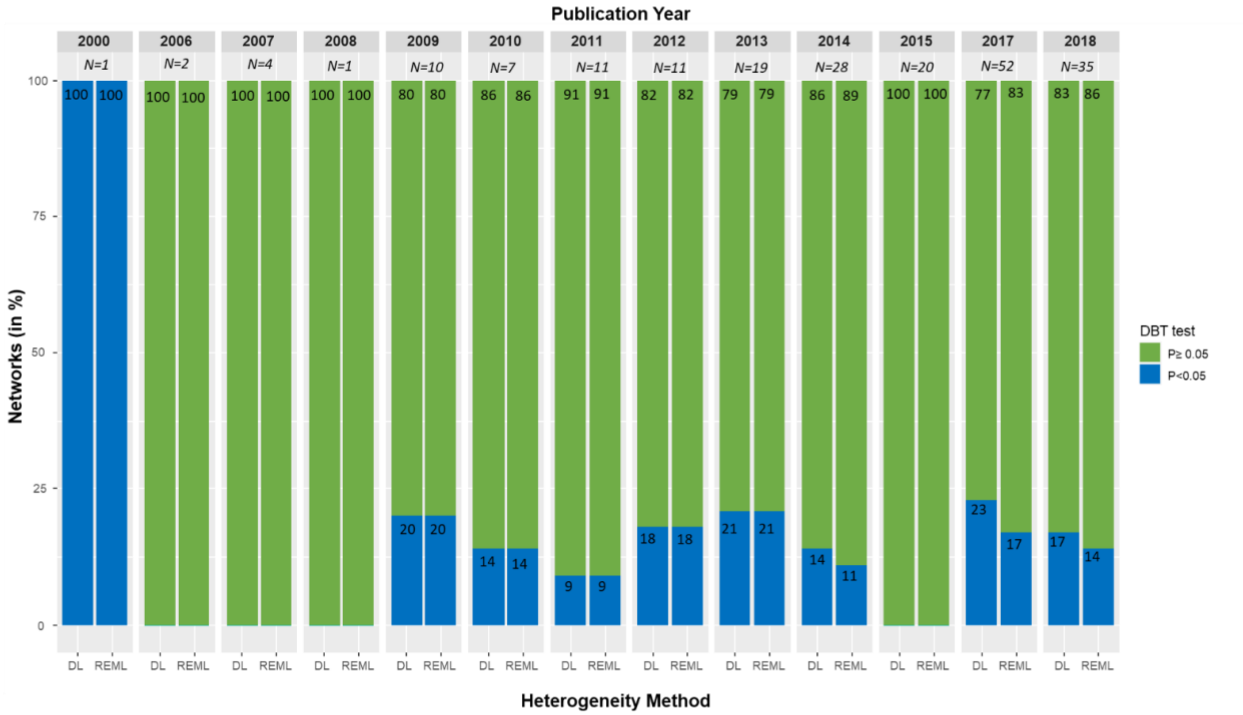

***** P is the p-value of the design-by-treatment inconsistency test

‡ NMAs published in the year 2016 were not included in this study

Chi-square test for the comparison of the inconsistency rates across years: p-value= 0.365 for DL and p-value= 0.385 for REML

Abbreviations: DL, DerSimonian and Laird; REML, restricted maximum likelihood

Appendix Figure 3: Plot of p-values (fourth-root scale) of the DBT model against network structural characteristics (logarithmic scale)

Panels (a) (correlation coefficient [p-value] = -0.10 [0.16]) and (b) (correlation coefficient [p-value] = -0.16 [0.02]) explore the number of studies in the network; panels (c) (correlation coefficient [p-value] = -0.03 [0.71]) and (d) (correlation coefficient [p-value] = -0.10 [0.16]) explore the number of interventions in the network; panels (e) (correlation coefficient [p-value] = -0.08 [0.26]) and (f) (correlation coefficient [p-value] = -0.16 [0.02]) explore the number of unique direct intervention comparisons in the network; panels (g) (correlation coefficient [p-value] = -0.03 [0.67]) and (h) (correlation coefficient [p-value] = -0.11 [0.11]) explore the number of designs in the network; panels (i) (correlation coefficient [p-value] = -0.02 [0.77]) and (j) (correlation coefficient [p-value] = -0.11 [0.10]) explore the number of multi-arm studies in the network; panels (k) (correlation coefficient [p-value] = 0.01 [0.67]) and (l) (correlation coefficient [p-value] = -0.05 [0.45]) explore the number of loops in the network. First column of panels uses the REML estimator for heterogeneity, whereas second column of panels uses the DL estimator for heterogeneity.

The horizontal green and purple lines represent the cut-off p-value = 0.05 and p-value = 0.10, respectively. The blue diagonal line is the regression line.

* Networks with no multi-arm studies in (i) and (j) plots were treated as networks including a single multi-arm study, to avoid excluding them from the plot, since the logarithm could not be calculated.

Abbreviations: DBT, design-by-treatment interaction model; DL, DerSimonian and Laird; REML, restricted maximum likelihood

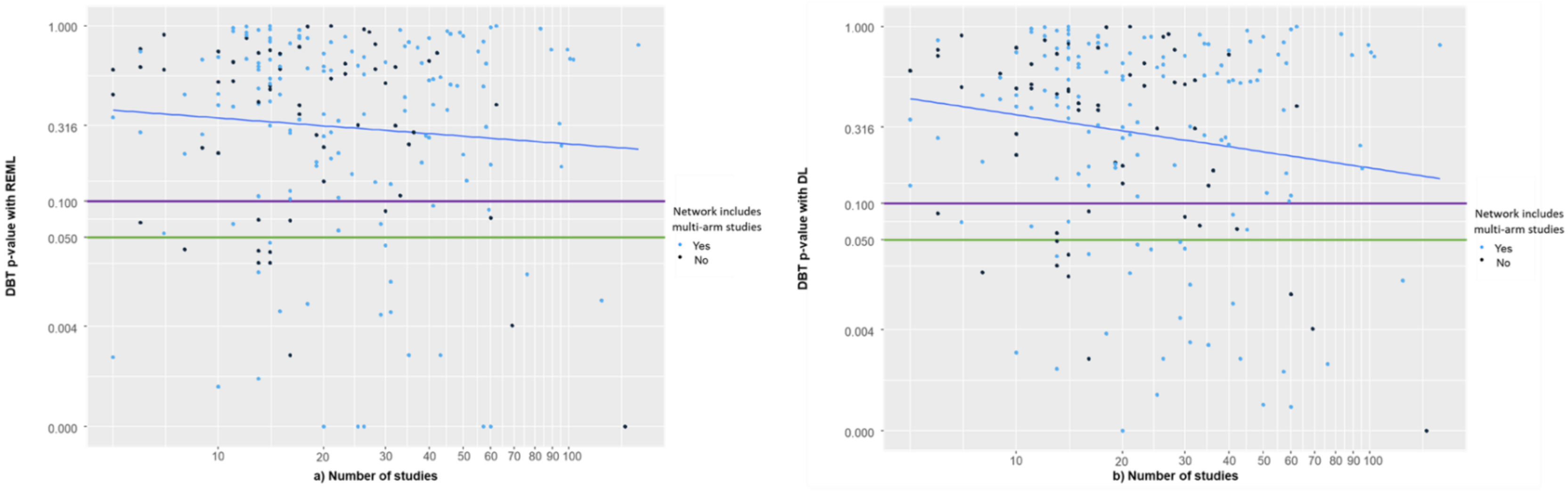

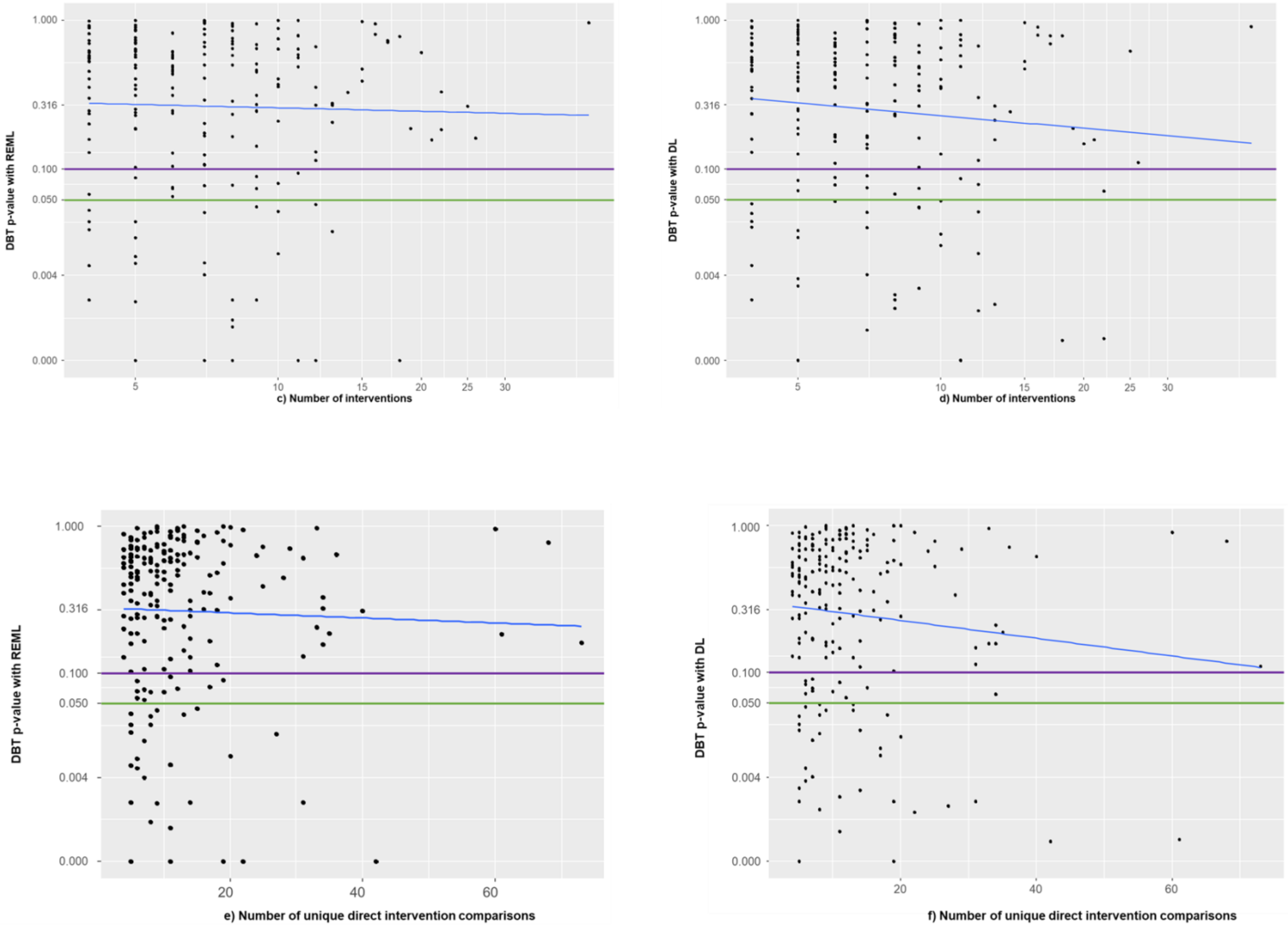

DBT p-value with REML
1.000
0.316
0.100
0.050
0.004
0.000
5
10
15
20
25
30
c) Number of interventions
DBT p-value with DL
d) Number of interventions
e) Number of unique direct intervention comparisons
20
40
60
f) Number of unique direct intervention comparisons

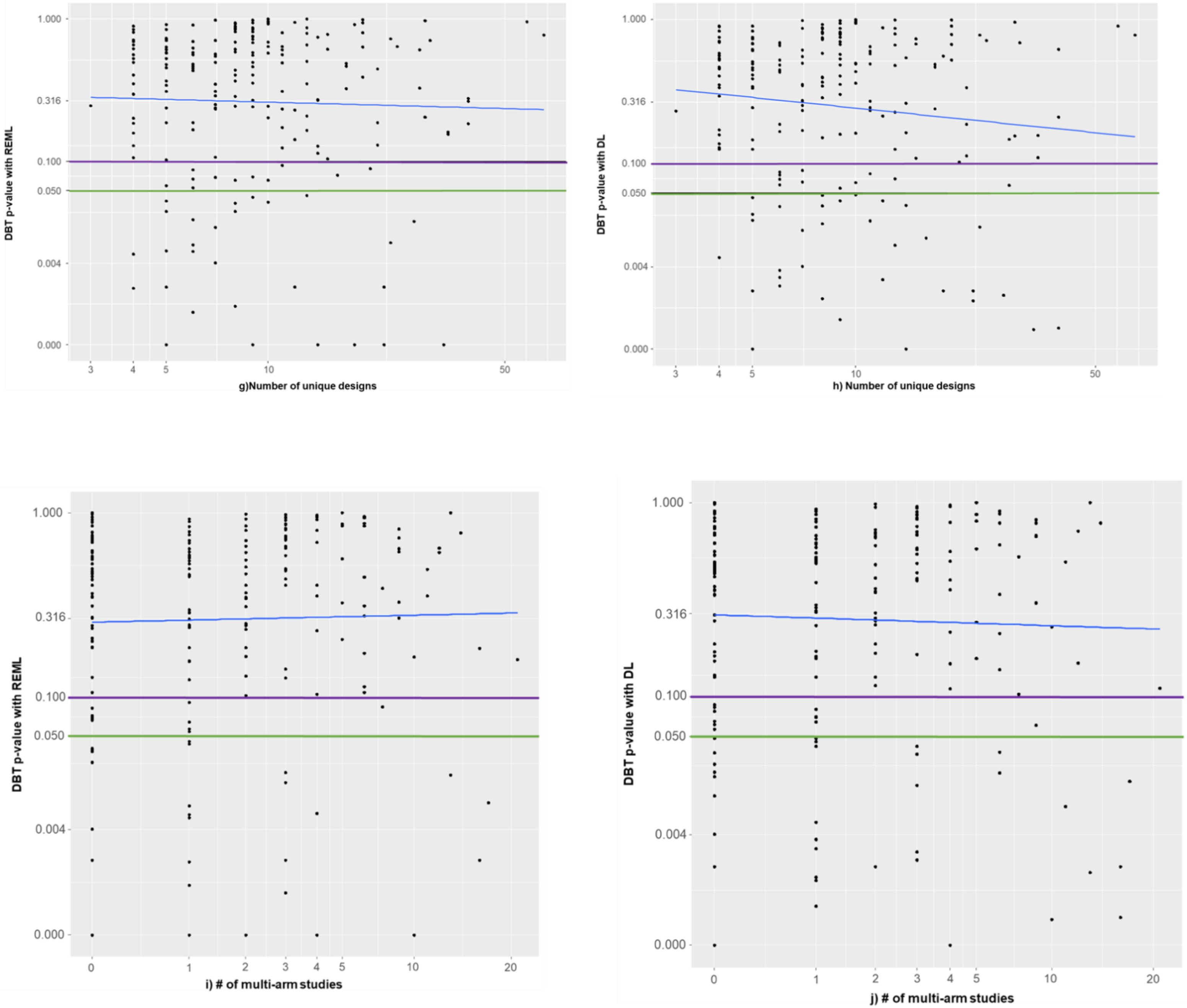
DBT p-value with REML
1.000
0.316
0.100
0.050
0.004
0.000
3
4
5
10
50
g)Number of unique designs
DBT p-value with DL
h) Number of unique designs
0
1
2
3
4
5
10
20
i) # of multi-arm studies
j) # of multi-arm studies

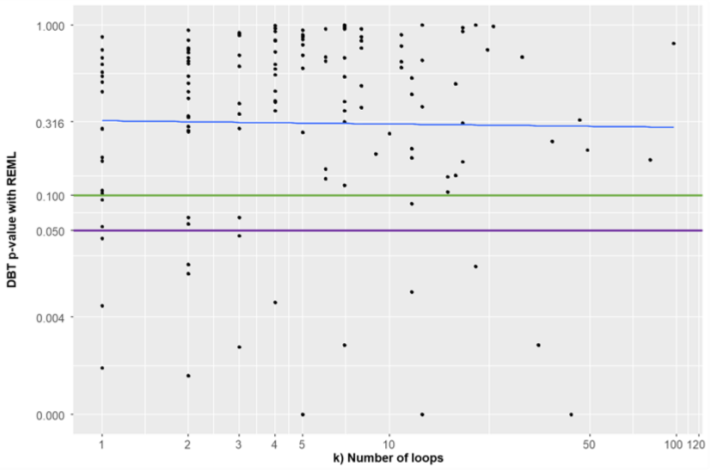
DBT p-value with REML
1.000
0.316
0.100
0.050
0.004
0.000
1
2
3
4
5
10
50
100 120
k) Number of loops

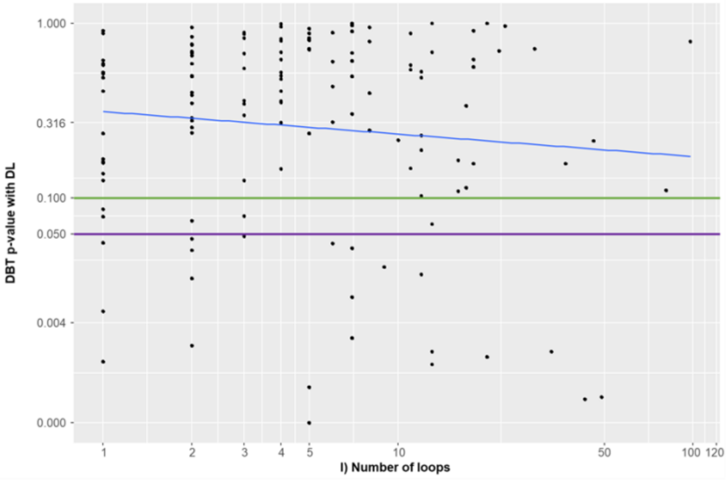
DBT p-value with DL
1.000
0.316
0.100
0.050
0.004
0.000
1
2
3
4
5
10
50
100 120
l) Number of loops

Appendix Figure 4: Plot of p-values (fourth-root scale) of the DBT model against ratios of network structural characteristics (logarithmic scale)

Panel (a) explores the ratio of the number of studies to the number of interventions in a network (correlation coefficient [p-value] = -0.07 [0.31]); panel (b) explores the ratio of the number of loops to the number of interventions in a network (correlation coefficient [p-value] = -0.01 [0.86]); panel (c) explores the ratio of the number of loops to the number of studies in a network (correlation coefficient [p-value] = 0.07 [0.31]); panel (d) explores the ratio of the number of designs to the number of studies in a network (correlation coefficient [p-value] = 0.07 [0.28]); panel (e) explores the ratio of the number of unique direct intervention comparisons to the number of studies in a network (correlation coefficient [p-value] = 0.09 [0.19]); panel (f) explores the ratio of the number of multi-arm studies to the number of total studies in a network (correlation coefficient [p-value] = 0.11 [0.12]). All analyses have used the DL estimator for heterogeneity.

The horizontal green and purple lines represent the cut-off p-value = 0.05 and p-value = 0.10, respectively. The blue diagonal line is the regression line.

* Networks with no multi-arm studies in (f) plot were treated as networks including a single multi-arm study, to avoid excluding them from the plot, since the logarithm could not be calculated.

Abbreviations: DBT, design-by-treatment interaction model; DL, DerSimonian and Laird

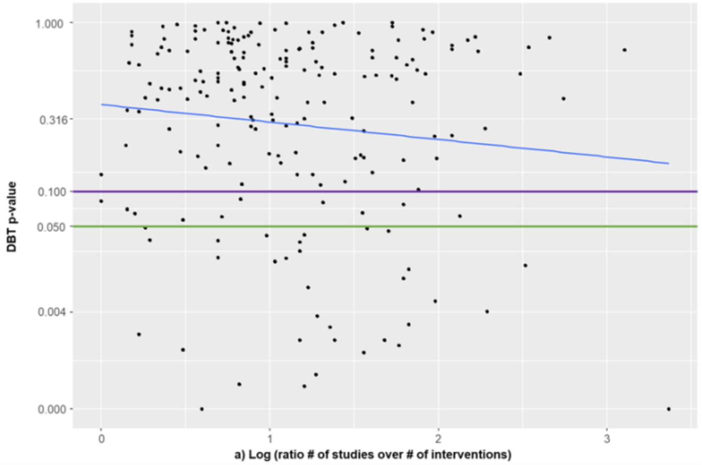

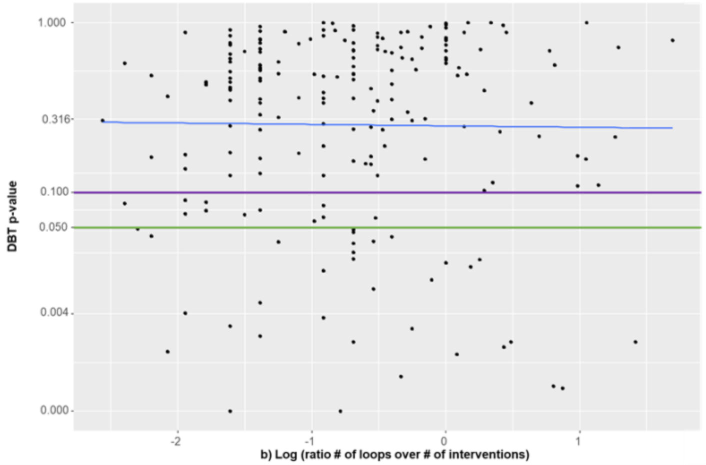

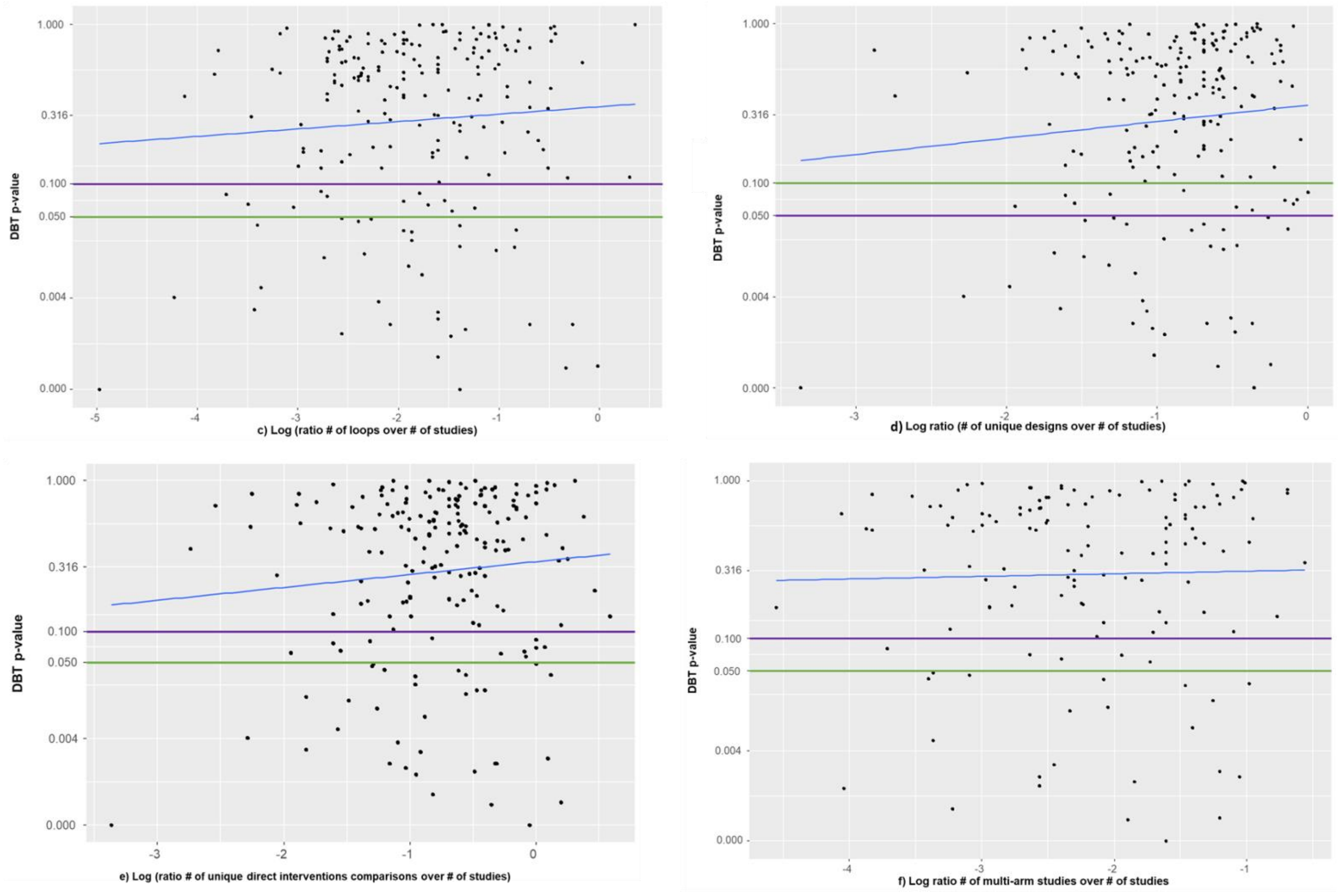

DBT p-value
1.000
0.316
0.100
0.050
0.004
0.000
-5
-4
-3
-2
-1
0
c) Log (ratio # of loops over # of studies)
d) Log ratio (# of unique designs over # of studies)
e) Log (ratio # of unique direct interventions comparisons over # of studies)
f) Log ratio # of multi-arm studies over # of studies

Appendix Figure 5: Plot of the between-study standard deviation estimated in the consistency model against the ratio of the number of studies to the number of interventions in a network (logarithmic scale)

The blue diagonal line is the regression line. Panel (a) represent the estimation of the between-study standard deviation with REML (correlation coefficient [p-value] = -0.06 [0.43]), whereas panel (b) with DL (correlation coefficient [p-value] = -0.05 [0.49]).

Abbreviations: DL, DerSimonian and Laird; REML, restricted maximum likelihood

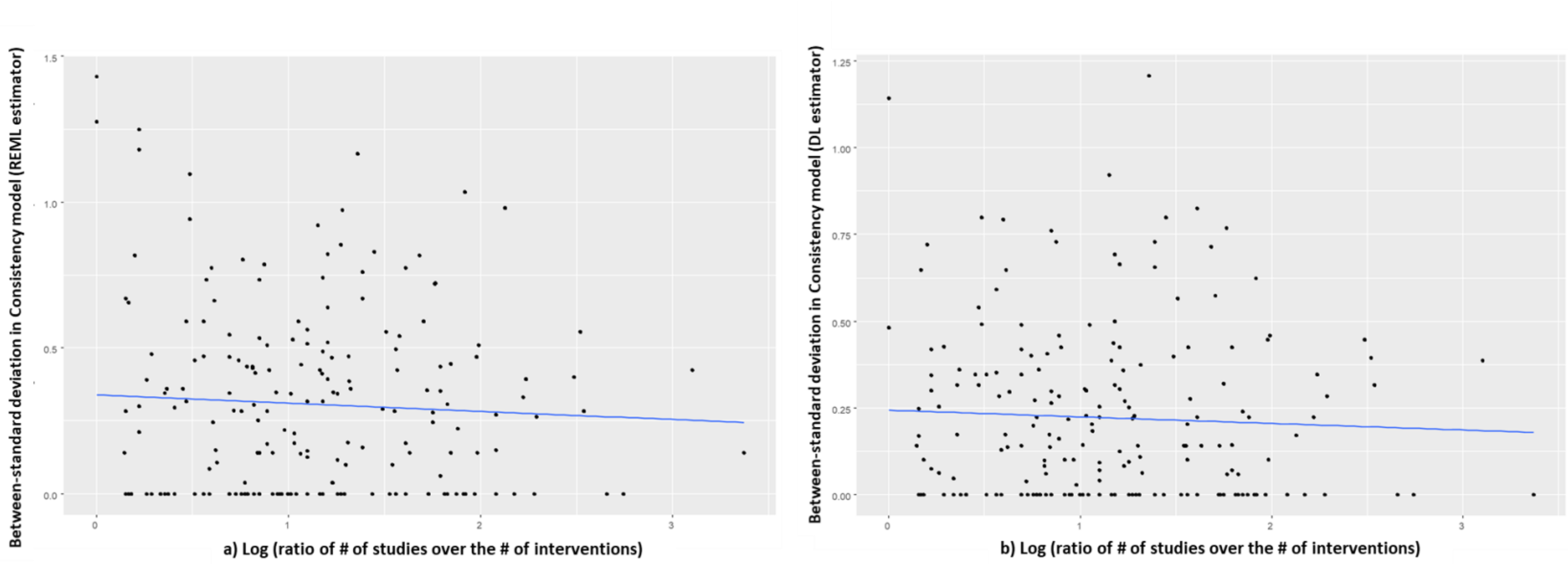

Appendix Figure 6: Box plot of the p-values (fourth-root scale) of the DBT model per (a) type of outcome, (b) type of intervention comparison, c) presence of complex interventions, and d) presence of at least one direct intervention comparison with a single study

The horizontal green and purple lines represent the cut-off p-value = 0.05 and p-value = 0.10, respectively. All analyses have used the DL estimator for heterogeneity.
Statistical assessment of difference in DBT p-values between groups: (a) p-value = 0.92, (b) p-value = 0.73, (c) p-value = 0.19, (d) p-value = 0.64

Abbreviations: DBT, design-by-treatment interaction model; DL, DerSimonian and Laird

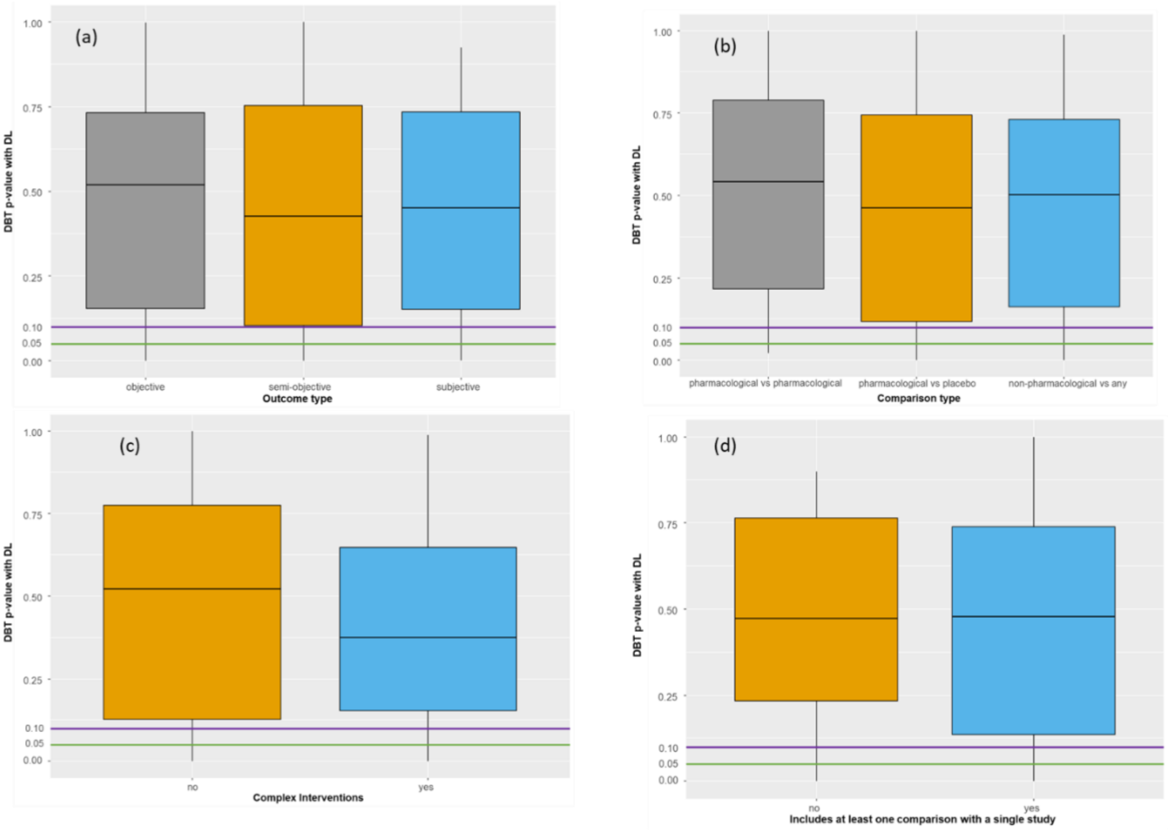

Appendix Figure 7: Plot of the p-values (fourth-root scale) of the DBT model against the I-squared

The horizontal green and purple lines represent the cut-off p-value = 0.05 and p-value = 0.10, respectively. The blue diagonal line is the regression line ((a) REML: correlation coefficient [p-value] = -0.38 [P<0.001]; (b) DL: correlation coefficient [p-value] = -0.38 [P<0.001]).

Abbreviations: DBT, design-by-treatment interaction model; DL, DerSimonian and Laird; REML, restricted maximum likelihood

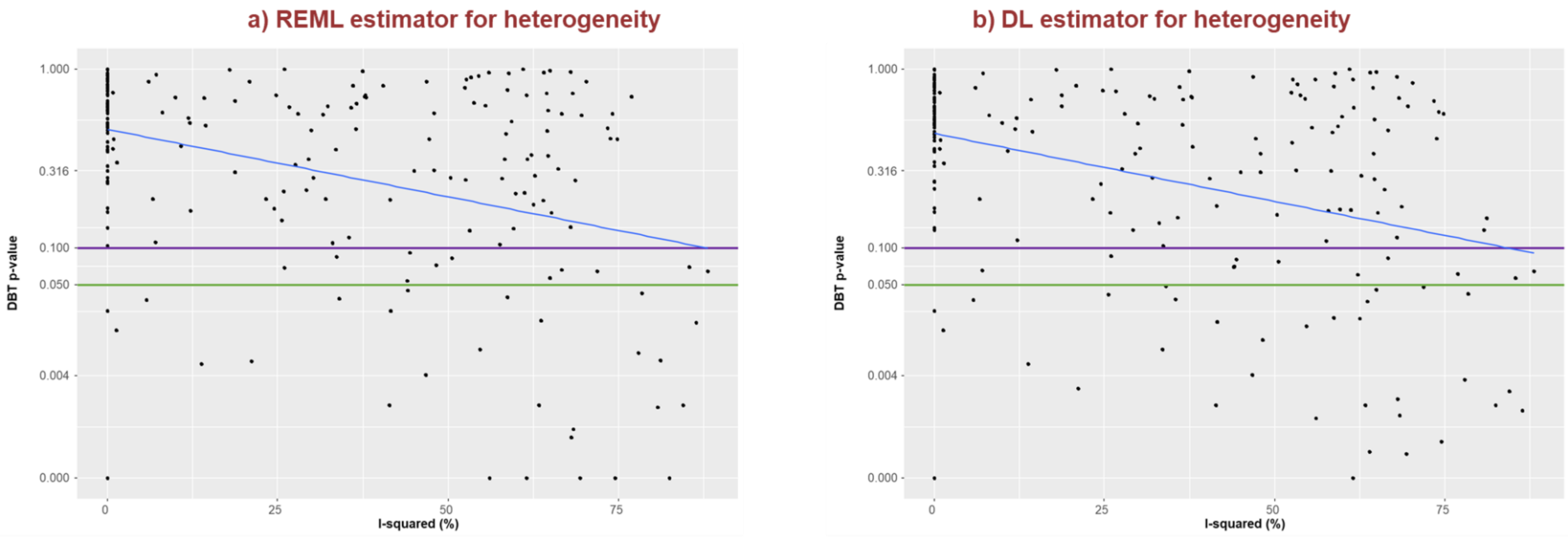

Appendix Figure 8: Plot of the between-study standard deviation in consistency against the inconsistency model

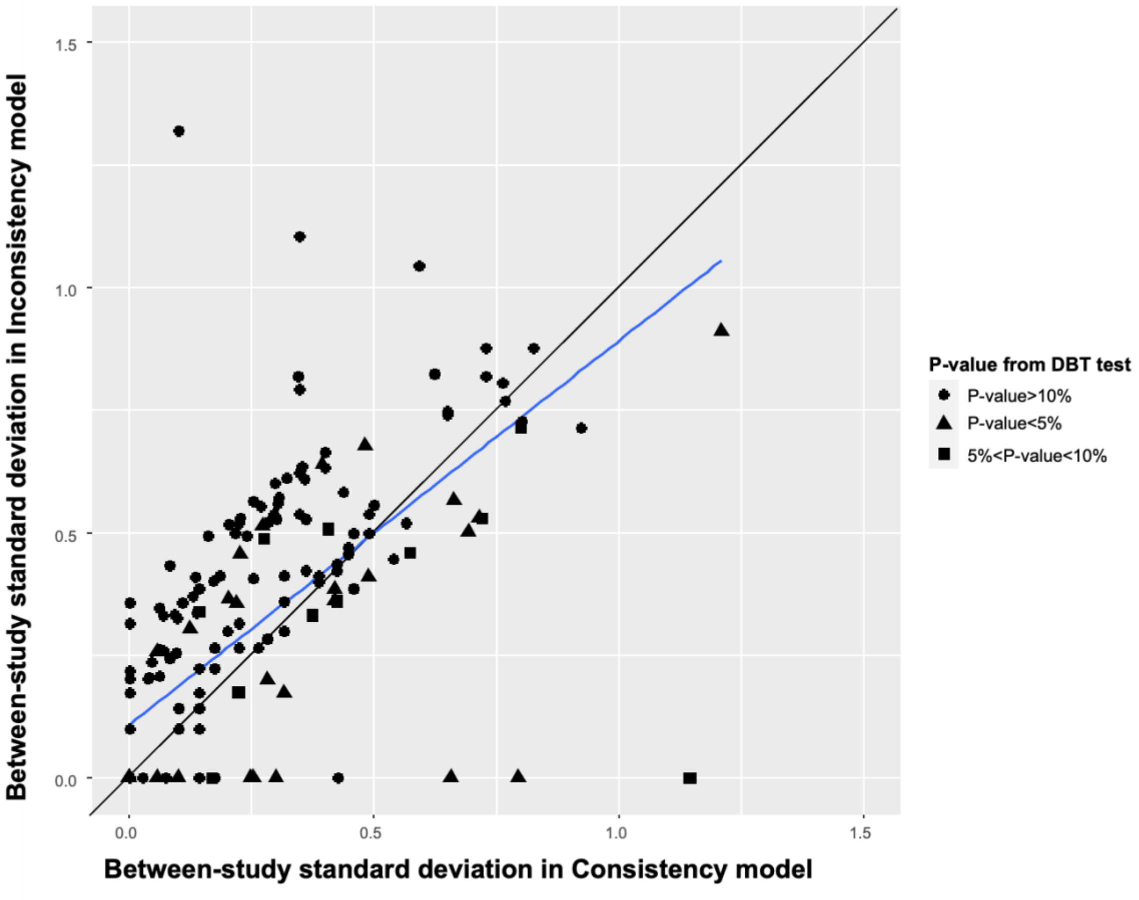

The black diagonal line represents equality in between-study standard deviation between consistency and inconsistency models.

Circle points represent the networks consistent at the significance level of 10%, triangular points represent the inconsistent networks at α=5%, and rectangular points represent networks inconsistent between the significance levels 5% and 10%.

All analyses have used the DL estimator for heterogeneity.

Abbreviations: DBT, design-by-treatment interaction model; DL, DerSimonian and Laird

Appendix Figure 9: Plot of the between-study standard deviation in inconsistency against the degrees of freedom of the Wald chi-square test (logarithmic scale)

Circle points represent the networks consistent at the significance level of 10%, triangular points represent the inconsistent networks at α=5%, and rectangular points represent networks inconsistent between the significance levels 5% and 10%.

Abbreviations: DBT, design-by-treatment interaction model; DL, DerSimonian and Laird; REML, restricted maximum likelihood

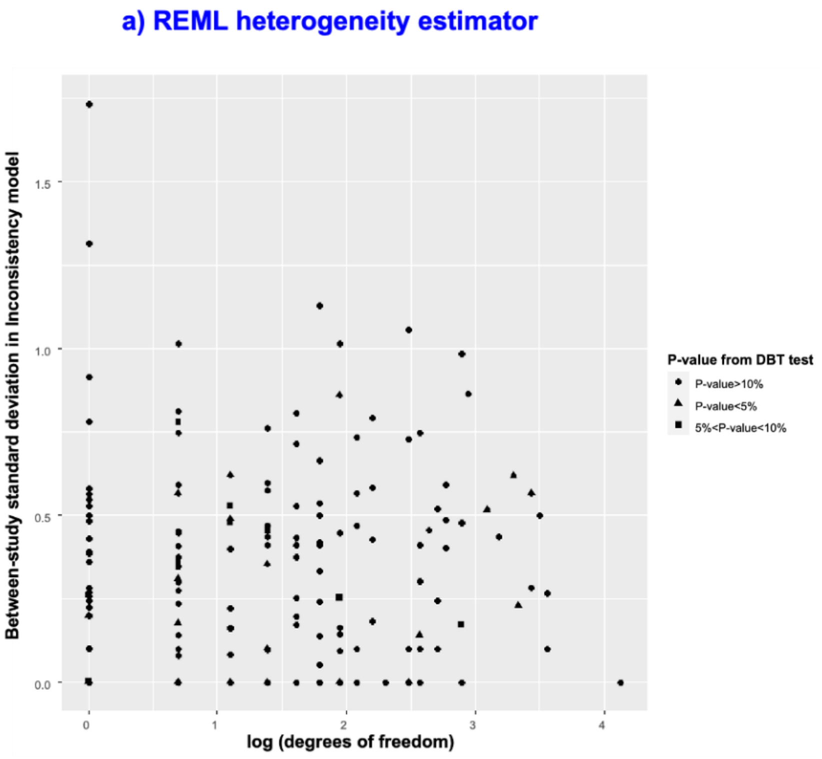

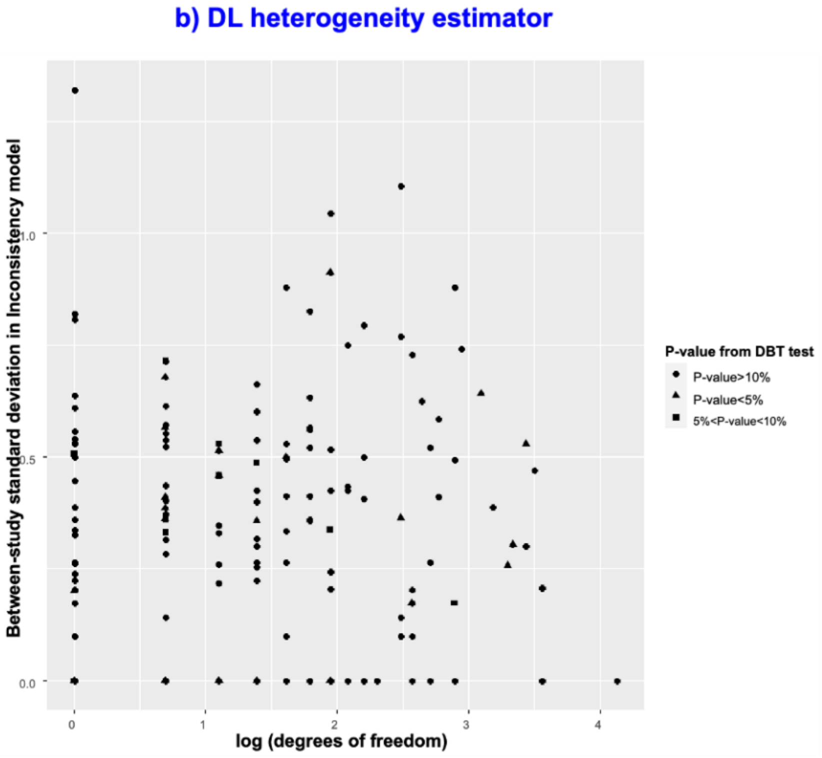

Supplement: Supplementary file 1 — Additional file 1: Appendix 1. Eligibility criteria, screening, study selection, and data abstraction. Appendix 2. Model description. Appendix Figure 1. Flowchart for network meta-analysis study inclusion. Appendix Table 1. Number of consistent and inconsistent networks at 0.05 and 0.10 significance levels using DL and REML heterogeneity estimators. Appendix Table 2. Multivariable regression analysis results. Appendix Figure 2. Stacked bar plot of consistent (green bars) and inconsistent (blue bars) networks at α = 0.05 per heterogeneity estimator and year of study publication‡. Appendix Figure 3. Plot of p-values (fourth-root scale) of the DBT model against network structural characteristics (logarithmic scale). Appendix Figure 4. Plot of p-values (fourth-root scale) of the DBT model against ratios of network structural characteristics (logarithmic scale). Appendix Figure 5. Plot of the between-study standard deviation estimated in the consistency model against the ratio of the number of studies to the number of interventions in a network (logarithmic scale). Appendix Figure 6. Box plot of the p-values (fourth-root scale) of the DBT model per (a) type of outcome, (b) type of intervention comparison, c) presence of complex interventions, and d) presence of at least one direct intervention comparison with a single study. Appendix Figure 7. Plot of the p-values (fourth-root scale) of the DBT model against the I-squared. Appendix Figure 8. Plot of the between-study standard deviation in consistency against the inconsistency model. Appendix Figure 9. Plot of the between-study standard deviation in inconsistency against the degrees of freedom of the Wald chi-square test (logarithmic scale). [file 12874_2021_1401_MOESM1_ESM.pdf]
